# Comparative analysis of transcriptome in oil biosynthesis between seeds and non-seed tissues of *Symplocos paniculata* fruit

**DOI:** 10.3389/fpls.2024.1441602

**Published:** 2024-10-02

**Authors:** Qiang Liu, Yunzhu Chen, Jingzhen Chen, Peiwang Li, Lijuan Jiang, Changzhu Li, Wenbin Zeng, Yan Yang

**Affiliations:** ^1^ College of Life and Environment Science, Central South University of Forestry and Technology, Changsha, China; ^2^ State Key Laboratory of Utilization of Woody Oil Resource, Hunan Academy of Forestry, Changsha, China

**Keywords:** *Symplocos paniculata*, oil biosynthesis, tissue specificity, transcriptome, gene expression patterns

## Abstract

The *Symplocos paniculata*, a woody oil plant, has garnered attention for its oil-rich fruit, which exhibits potential for both oil production and ecological restoration endeavors, thereby presenting substantial developmental value. However, the comprehension of the distinctive oil biosynthesis and deposition strategies within the fruit’s various compartments, coupled with the tissue-specific biosynthetic pathways yielding optimal fatty acid profiles, remains in its infancy. This investigation was designed to delineate the tissue specificity of oil biosynthetic disparities and to elucidate the molecular underpinnings within the fruit mesocarp and seeds of *S. paniculata*, employing lipidomic and transcriptomic analyses. The results revealed that oil biosynthesis within the fruit mesocarp commences approximately 40 days prior to that within the seeds, with a concomitant higher lipid content observed in the mesocarp, reaching 43% as opposed to 30% in the seeds. The fruit mesocarp was found to be enriched with palmitic acid (C16:0) and exhibited a harmonious ratio of saturated, monounsaturated, to polyunsaturated fatty acids (SFA: MUFA: PUFA=1:1:1), in stark contrast to the seed oil, which is predominantly composed of unsaturated fatty acids, accounting for 90% of its total FA content. Microstructural assessments have unveiled divergent oil deposition modalities; the fruit mesocarp oils are predominantly sequestered within oil cells (OC) and a spectrum of lipid droplets (LD), whereas the seeds predominantly harbor uniformly-sized LD. The expression patterns of pivotal genes implicated in oil biosynthesis were observed to be markedly contingent upon the tissue type and developmental stage. Notably, the light-responsive fatty acid synthase (FAS) gene demonstrated preferential transcription within the fruit mesocarp. In contrast, genes pivotal for carbon chain elongation, such as 3-ketoacyl-ACP synthase II (KASII) and fatty acyl-ACP thioesterase A (FATA), and desaturation, typified by Stearoyl-ACP desaturase (SAD) and Fatty Acid Desaturase (FAD), were noted to be more robustly transcribed within the seeds. Furthermore, isoenzyme gene families integral to the assembly of triacylglycerol (TAG), including long-chain acyl-CoA synthetases (LACSs), glycerol-3-phosphate acyltransferases (GPATs), and lysophosphatidic acid acyltransferases (LPATs), exhibited pronounced tissue specificity. This research endeavors to clarify the molecular regulatory mechanisms that oversee oil biosynthesis within both seed and non-seed tissues of oilseed-bearing plants with entire fruits. Collectively, these findings lay the groundwork and offer technical scaffolding for future targeted cultivation of woody oil plants, with the ultimate aim of augmenting fruit oil yield and refining FA compositions.

## 1 Introduction

The *Symplocos paniculata*, a member of the esteemed *Symplocaceae* family, is an oilseed producing plant of immense ecological and economic significance originating from the enchanting lands of eastern Asia, encompassing China, Japan, and Korea (Liu et al., 2016). Due to its high oil content and exceptional fatty acid composition, the fruit oil of *S. paniculata* has been identified as a promising source for culinary oil, biodiesel production (Liu et al., 2011), as well as various industrial applications such as ink surfactants, lubricants, and soap (Guan, 1991). Moreover, it plays a pivotal role in preserving ecosystem functions by effectively mitigating desertification and erosion due to its remarkable tolerance towards severe drought, high salinity, and alkalinity in soils (Guan, 1991). Furthermore, the naturally stunted growth of *S. paniculata* implies minimal management requirements for commercial-scale cultivation. However, the scientific and economic importance of this species is not matched by the available literature and molecular resources, which remain limited.

Similar to avocado (*Persea americana*), oil palm (*Elaeis guineensis*), and olive (*Olea europaea*), *S. paniculata* accumulates copious amounts of oil in both the seed and mesocarp of its fruit. As we are cognizant, the oil stored within seeds serves as a catalyst for post-germinative growth of seedlings, while mesocarp oil functions as an enticing lure for disseminating animals and remains unaltered by subsequent plant metabolism. Nevertheless, both oils are predominantly comprised of triacylglycerols (TAGs), which consist of glycerol esterified with three fatty acids (FAs). The versatile applications of vegetable oils are primarily determined by the fatty acid composition of triacylglycerols. The mesocarp oil derived from *S. paniculata* is rich in palmitic acid (C16:0), making it an excellent choice for soap production and other industrial applications. Conversely, seed oil with a high proportion of oleic (C18:1) and linoleic acid (C18:2) is highly recommended for culinary purposes (Li et al., 2023). Moreover, notable disparities in the relative proportions of fatty acids between seed and non-seed tissues (primarily mesocarp) have been observed in other oil-producing plants (Tranbarger et al., 2011; Turesson et al., 2010). Currently, our comprehension of TAG accumulation in plants primarily stems from investigations on oil seeds (Perez de Souza et al., 2020). However, given the ever-growing global demand for vegetable oils utilized both in culinary and industrial applications, it is imperative that we discern the factors governing these quantitative and qualitative variations. Henceforth, greater emphasis should be placed on those plant species that store oil in tissues other than seeds.

The economic value of this woody oil plant is often determined by the fruit oil content and quality, as it serves as the primary metabolite of *S. paniculata*. Therefore, comprehending the biosynthesis pathway and regulatory mechanism of oil is imperative in order to enhance its content and composition in both seed and non-seed tissues (Zhang et al., 2019; Ding et al., 2017). The intricate process of oil biosynthesis has been extensively demonstrated by numerous studies, encompassing the intricate distribution of carbon sources and involving pathways such as glycometabolism, fatty acid biosynthesis, and triacylglycerol assembly (Lin et al., 2017). However, it is worth noting that the regulation of key gene expression exhibits tissue-specificity in fruits and undergoes variations throughout different developmental stages (Takenaga et al., 2008). Moreover, transcriptome studies conducted on plants, including avocado (*Persea americana*) and oil palm (*Elaeis guineensis*), have unveiled substantial disparities in the transcriptional control of oil biosynthesis between non-seed tissues and seed tissues. The heightened expression levels of FATA and FAD2 genes in avocado (*Persea americana*) seeds, for example, may mirror the enhanced lipid composition of C18:2 and C18:3 in comparison to their mesocarp (Kilaru et al., 2015). Oil palm (*Elaeis guineensis*) has the ability to accumulate up to 90% of its weight as oil due to the highly expressed Diacylglycerol O-acyltransferase 1 (DGAT1) and DGAT2 genes in its mesocarp, surpassing that found in seeds (Dussert et al., 2013).

In an endeavor to elucidate the unique characteristics underlying the elevated lipid biosynthesis in both the seed and non-seed tissues of *S. paniculata* fruits, a comprehensive comparative analysis of the transcriptome and metabolite profiles was executed between the seed and mesocarp during the developmental stages of the fruit. Furthermore, to delve deeper into this phenomenon, lipid droplet analyses were conducted, augmented by electron microscopy techniques. The transcriptome dataset generated from this study is anticipated to constitute a valuable asset, which could substantially aid in the biotechnological improvement of both seed and non-seed biomass in *S. paniculata*. This advancement is likely to have profound implications for the enhancement of biofuel and cooking oil production, potentially transforming the landscape of sustainable energy and food industries.

## 2 Materials and methods

### 2.1 Plant materials

The collection of fresh *Symplocos paniculata* fruits was meticulously conducted at an interval of 10 days after flowering (DAF) throughout the year 2022, sourced from the Hunan Academy of Forestry, located at 28°06′ 55″ N latitude and 113° 03′16″ E longitude, in Changsha city, Hunan Province, China. Each developmental phase was represented by a homogeneous pool of fruits, which were systematically apportioned into four distinct portions for subsequent analysis: transcriptome sequencing, oil extraction, and microstructure examination. Specifically, one portion of the samples underwent rapid cryopreservation in liquid nitrogen subsequent to the separation of seeds and peels, encapsulated in aluminum foil, and subsequently preserved at −80°C for deferred analysis. Another portion was preserved in FAA fixative (a mixture of formaldehyde, acetic acid, and ethyl alcohol, volume ratio 5:5:90) and used to create frozen sections for microscopic observation. The remaining samples were segregated into their respective pulp and seed components, subjected to a drying process at 60°C for a duration of four days until a consistent weight was achieved. These were then pulverized using an RT-02 miniature plant pulverizer and subsequently sieved through a series of stainless steel meshes to achieve a uniform particle size of 0.15 mm.

### 2.2 Methods

#### 2.2.1 Oil content determination

The oil content of the fruit was determined in accordance with Chinese national standard methods GB/T 5512-2008 (Liu et al., 2016). A precise quantity of 4 grams each of the desiccated and pulverized peels and seeds was thoroughly combined with 60 mL of high-purity petroleum ether (99.7% purity, with a boiling point range of 30 to 60 °C) within an SZE-101 Fat Analyzer, manufactured by Shanghai ShineJan Instruments Co. Ltd., Shanghai, China. The resultant mixture was subjected to heating under reflux conditions at a controlled temperature of 65 °C for a duration of six hours, accompanied by intensive mechanical stirring. Subsequently, the mixture underwent filtration and was then subjected to evaporation under reduced pressure using a rotary vacuum evaporator, maintained at a temperature of 62.5 °C, to obtain the oil. Post-extraction, the samples underwent a further desiccation phase in an oven, set to a constant temperature of 110 °C, for a period of four hours, until a stable mass was achieved. The final recorded mass was utilized to ascertain the oil content (w) through the application of the following formula: Oil content (%) = (Ma - Mb)/Ma × 100%. In this formula, Ma corresponds to the initial mass of the sample prior to the extraction process, and Mb is indicative of the sample’s mass after the completion of the extraction, with a measurement precision of 0.0001 grams.

#### 2.2.2 Fatty acid composition determination

The Fatty acid components of the fruit oil were analyzed using a Clarus 600 gas chromatograph-mass spectrometer (GC-MS, Perkin Elmer Instrument Co., Ltd, Shanghai, China). The saponification of 30 g of mesocarp or seed oil was carried out in a 0.5 mol·L^-1^ blending solution, which was prepared by adding 28.1 grams of potassium hydroxide (KOH) to one liter of methyl alcohol (99.9%). The resulting saponified fruit oil was separated into layers by the addition of 10 mL each of petroleum ether (99.9%) and deionized water, followed by centrifugation at 3000 r·min^-1^ for 5 minutes using a refrigerated centrifuge model 3H16RI from Hunan Hexi Instruments Co., Ltd., Chang Sha, China. The supernatant was injected into a 0.3 mm×25 m free fatty acid polyester (FFAP) column. The identification of the fatty acid components was conducted using the Wiley mass spectral library. The relative percentage of fatty acid compositions was determined based on the peak areas (Li et al., 2023).

#### 2.2.3 Microscopic observation

The harvested fruits were initially fixed using a formalin-acetic acid-alcohol (FAA) solution to preserve their cellular structure. Following fixation, the specimens were meticulously washed to remove any residual fixative and subsequently immersed in a glycerol solution. A vacuum infiltration process was applied to facilitate the thorough infiltration of glycerol into the interstitial spaces of the fruit tissues. For the preparation of cross-sections, a cryostat microtome was utilized, set at a low temperature of -30°C to maintain the integrity of the samples during sectioning. The fruit samples were rapidly cryofixed for a brief period of 30 seconds, yielding thin sections with a uniform thickness of 25 µm, which were then mounted as temporary slides (Wang et al., 2012). These slides were subsequently stained using Sudan III for lipid detection and K-I staining solution to visualize cellular structures, with an incubation period of 20 min. The stained slides were examined under a Motic digital microscopy system to assess the microstructure and oil distribution (Er et al., 2000).

#### 2.2.4 Transcriptome sequencing and analysis

RNA was meticulously extracted from both mesocarp and seed tissues of *S. paniculata* fruits using a nucleic acid purification kit provided by Sangon Biotech., Shanghai, China. The spectrophotometric assessment of the RNA’s purity and concentration was conducted employing a Nanodrop 2000 ultra microspectrophotometer. Thereafter, the complementary DNA (cDNA) library was synthesized following the protocols outlined in the mRNA-Seq sample preparation kit by Illumina Biotechnology Company, United States, culminating in sequencing on the HiSeq 2500 platform, an Illumina technology. The sequencing endeavors were undertaken by Guangzhou Gidio Biotechnology Co., Ltd. Stringent quality control measures were implemented using the Fastp software suite (version 0.23.1) (Chen et al., 2018), ensuring the acquisition of high-fidelity data subsequent to the exclusion of substandard sequences. The assembled reads were generated via the Trinity assembler (version 2.1.1) (Grabherr et al., 2011), and the functional annotation was executed by aligning the sequences to the KEGG database, a comprehensive public repository. The quantification analysis was performed employing the RSEM tool (Li and Dewey, 2011). For the gene differential expression analysis, the input data comprised read count data derived from gene expression profiling. A comparative analysis of key enzymatic genes implicated in sugar metabolism and lipid biosynthesis within the mesocarp and seed tissues of *S. paniculata* was conducted, utilizing the Fragments Per Kilobase of transcript per Million mapped reads (FPKM) values. The gene expression ratio, defined as the FPKM ratio of Mesocarp to Seed (M/S), served as a metric to assess the differential gene expression between these two tissue types.

#### 2.2.5 qRT-PCR Verification

Total RNA was extracted from mesocarp and seed tissues of *S. paniculata* at various developmental stages. This RNA was reverse transcribed into first-strand cDNA using the SYBR Premix Ex Taq Kit (TaKaRa, Mountain View, CA, USA). We selected three key genes from each tissue type (six genes in total), known to be involved in lipid biosynthesis: ACC, PDAT1 and DGAT2. Primers for these genes were designed using Primer Premier 5.0 (Premier Biosoft International, Palo Alto, CA, USA), with sequences listed in **Supplementary Table S1**. ACTR3 (actin-related protein 3) and 18S rRNA served as reference genes for qPCR, with triplicate reactions for each target gene. The qRT-PCR protocol included an initial denaturation at 95°C for 2 minutes, followed by 40 cycles of 95°C for 15 seconds, and annealing/extension at 60°C for 15 seconds, with a final extension at 95°C for 15 seconds. The relative gene expression levels were calculated using the comparative cycle threshold method (ΔΔCt). This method involves comparing the Ct values of target genes to those of housekeeping genes for both the test and control groups. The ΔΔCt value and the FPKM value of the target unigene at 10 DAF were set to 1, and the expression levels at other time points (50, 90, 130, and 170 DAF) were determined as multiples of the expression at 10 DAF.

## 3 Results

### 3.1 The tissue specific pattern of oil accumulation between mesocarp and seed

**Figure 1:**
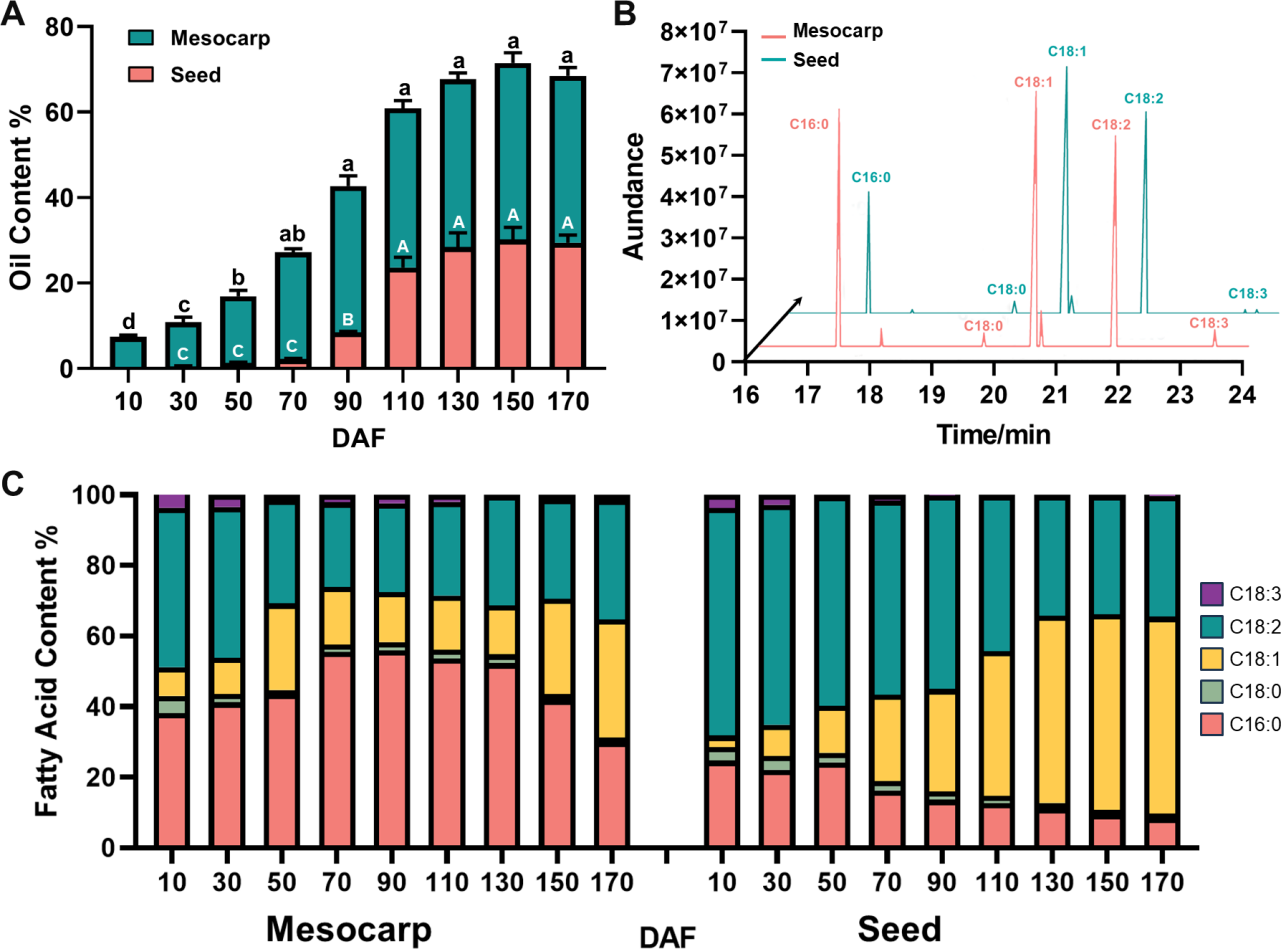
Changes of oil content and fatty acid composition of *S. paniculate* fruit. **(A)**, Oil content changes in mesocarp and seed; **(B)**, The main fatty acid components in mesocarp and seed (170 DAF); **(C)**, The dynamic changes of fatty acids’ relative content in the mesocarp and seed. Letters indicate differences between developmental stages by Tukey’s honestly significant difference multiple comparison at P < 0.05.

During the fruit developmental process of *S. paniculata*, there was a remarkable “S”-shaped alteration in the accumulation of oil within both the mesocarp and seeds. Specifically, oil content within the mesocarp began to accumulate at 10 DAF and reached a maximum of 43% at fruit maturity (150 DAF). Oil accumulation in seeds occurred later than that in mesocarp, with a small amount of oil beginning to accumulate at 50 DAF. Subsequently, seed oil content increased rapidly from 70 DAF to 110 DAF, followed by a slow increase from 140 DAF until reaching the maximum (about 30%) at 170 DAF and remaining stable thereafter (**Figure 1A**).

The mesocarp and seed oils of *S. paniculata* displayed a remarkable resemblance in their fatty acid composition, predominantly consisting of saturated fatty acids (SFA) such as C16:0 and stearic acid (C18:0), alongside monounsaturated fatty acid (MUFA) of C18:1, polyunsaturated fatty acid (PUFA) of C18:2 and linolenic acid (C18:3) as illustrated in **Figure 1B** (**Supplementary Figures S1**, **S2**). The lipid composition of *S. paniculata*’s mesocarp and seed displayed tissue-specificity, characterized by an augmentation in the proportion of palmitic acid and oleic acid in the former, accompanied by a reduction in linoleic acid. In mature fruit, the content ratio of SFA: MUFA: PUFA in mesocarp was approximately balanced at 1:1:1. During the development of *S. paniculata* fruit, the proportion of linoleic acid and palmitic acid components gradually diminishes while the relative content of oleic acid progressively increases. Upon maturation, the relative content of oleic acid reaches an impressive peak at 56%, with total unsaturated fatty acids accounting for a remarkable 90% (**Figure 1C**). The storage mode and component proportion of oil in various parts of *S. paniculata* fruit exhibit remarkable disparities.

### 3.2 The disparity in oil storage between seed and mesocarp tissue

**Figure 2:**
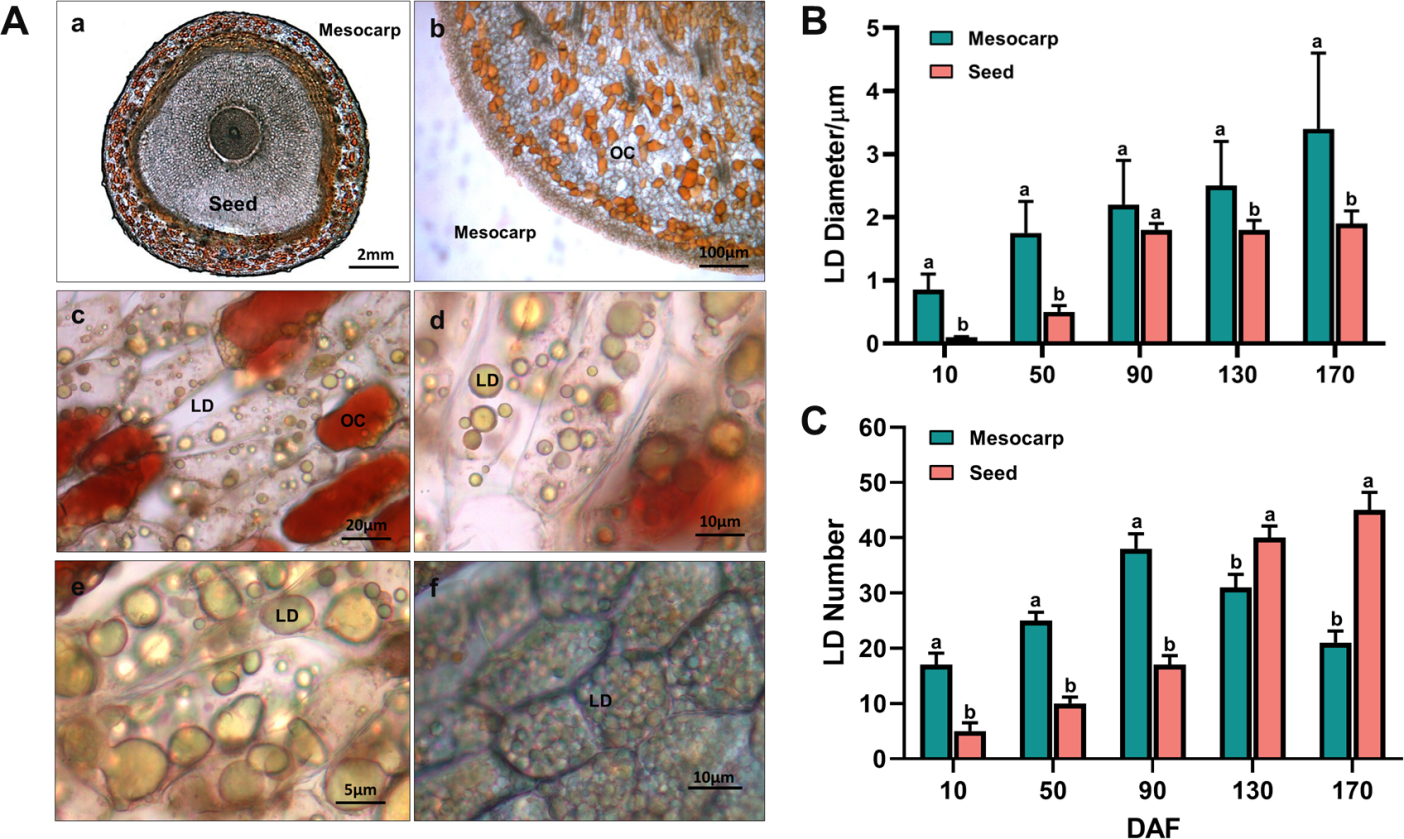
Dynamic change of oil cells (OCs) and lipid droplets (LDs) in the fruit of *S. paniculata.* **(A)**, The morphological variations of OCs and LDs in170 DAF (a, transverse section of entire fruit; b, the OCs in mesocarp; c, the OCs and LDs in mesocarp cell; d-e, the LDs in mesocarp cell; f, LDs in seed cell); **(B)**, The temporal fluctuations of LD size; **(C)**, The temporal fluctuations of LD number. Lowercase letters indicate differences between mesocarp and seed by Student’s t test at P < 0.05.

Transverse section images (**Figure 2A-a**) reveal that the entire fruit of *S. paniculata* contains oil, predominantly stored in the form of Oil Cells (OC) and Lipid Droplets (LD). In the mesocarp, oil is mainly stored in OC and LD, while in the seeds, it is primarily stored within intracellular LD. The OCs are distributed either randomly or in clusters within the mesocarp’s parenchyma tissue, exhibiting irregular shapes and sizes (**Figure 2A-b**), and their color deepens to a reddish hue with increasing oil accumulation (**Figure 2A-c**). Initially, small LDs accumulate within the mesocarp cells, which subsequently fuse to form larger LDs, resulting in a reduced number and size variation of LDs by maturity (**Figure 2A-de**). LD accumulation in the seeds commences later than in the mesocarp, starting at 90 DAF, with rapid development, increased volume, and a rise in quantity, culminating in a uniform distribution throughout the cells in a spherical form (**Figure 2A-f**). The largest LDs are found within the mesocarp cells, with an average diameter of 3.4μm (**Figure 2B**). The seeds contain the highest number of LDs, averaging 44 per cell (**Figure 2C**). Overall, significant differences in lipid storage mechanisms are observed across various regions of the *S. paniculata* fruit, which may account for the observed variations in lipid content and fatty acid composition.

### 3.3 Transcriptome sequencing analysis and functional annotation of *S. paniculata* fruit

A plethora of research has demonstrated that the pathways for oil synthesis in both seeds and non-seed tissues are generally analogous across most oleaginous woody plants. To delve deeper into the intricate mechanism of lipid synthesis in *S. paniculata* fruit, we employed cutting-edge second generation high-throughput transcriptome sequencing RNA-Seq technology to conduct a comparative bioinformatics analysis of the mesocarp and seeds at five distinct developmental stages. This was further complemented by leveraging public protein database BLAST comparison and KEGG Pathway functional group annotation.

**Figure 3:**
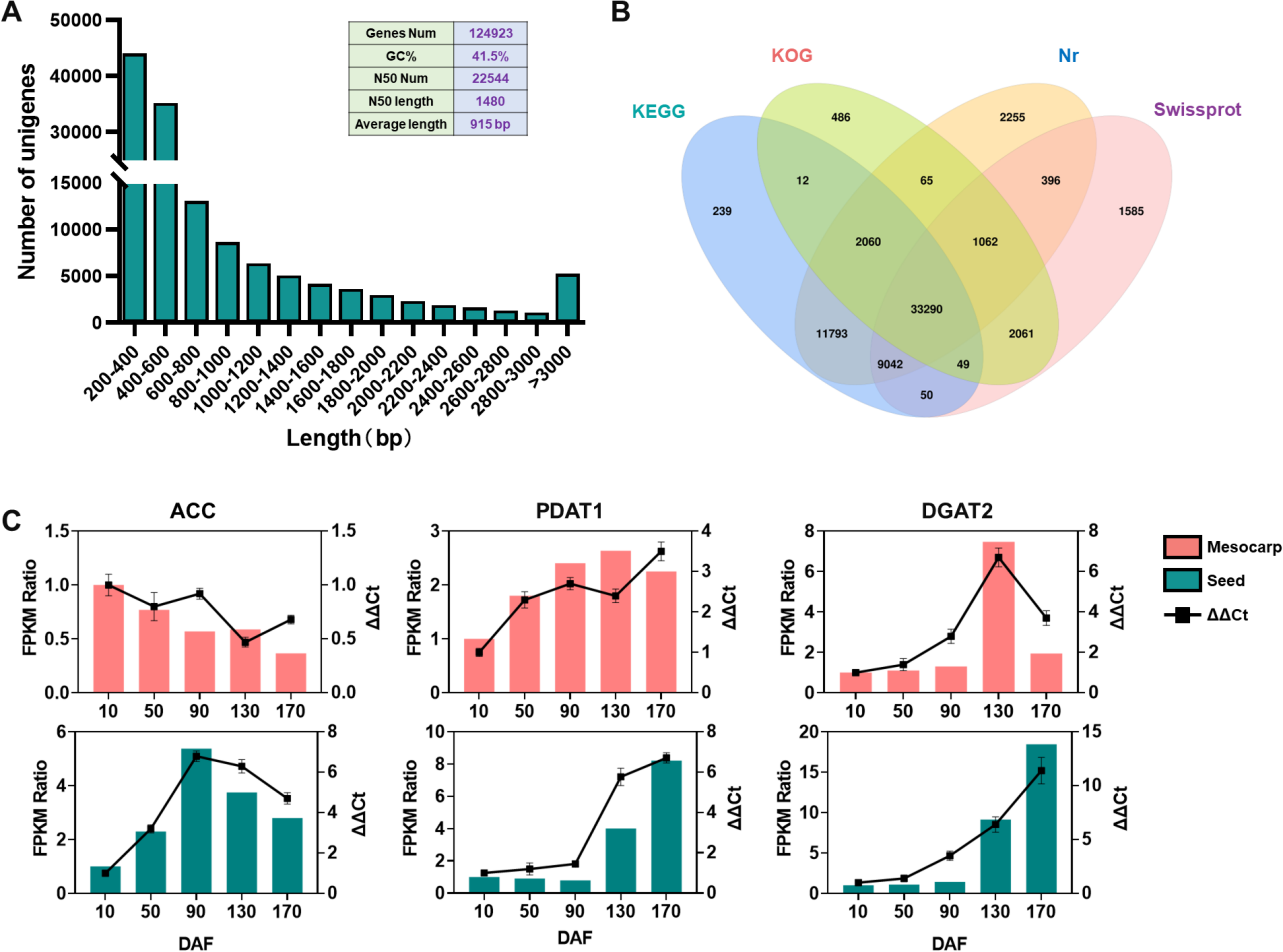
Transcriptome sequencing analysis and functional annotation of genes across different tissues of the *s. paniculata* fruit. **(A)**, Frequency Analysis of unigenes in *S. paniculata* fruit; **(B)**, Comparative Venn Diagram Analysis of unigene functional annotation across multiple public protein databases; **(C)**, Quantitative RT-PCR validations of the candidate oil biosynthesis genes in mesocarp and seeds of *Symplocos paniculate* fruit.

Transcriptome sequencing of *S. paniculata* ‘s mesocarp and seeds across various developmental phases yielded 114,404,745 assembled bases using the Trinity software, culminating in the identification of 124,923 non-redundant unigenes with an average length of 915 base pairs (bp), a GC content of 41.5%, and an N50 value of 1,480bp, indicative of extended sequence lengths. Collectively, these metrics attest to the high quality of the assembly. Analysis of the unigene length distribution revealed a gradual decline in the number of unigenes with increasing length, maintaining a consistent and smooth trend, further substantiating the sequencing results’ excellence (**Figure 3A**). BLAST alignment against the KEGG, KOG, Nr, and Swissprot databases identified 56,535, 39,085, 59,963, and 47,535 unigenes, respectively, with 33,290 genes annotated across all databases (**Figure 3B**). Comparative annotation of unigenes from the mesocarp and seeds at different developmental stages was mapped onto GO (**Supplementary Figure S3**) and KEGG **Supplementary Figure S4**) enrichment pathways, providing in-depth insights into the genes’ involvement in cellular processes and their functional roles. To rigorously validate the fidelity and accuracy of our RNA-Seq data, we meticulously curated a cohort of six genes pivotal to lipid biosynthesis, with an equal distribution from both fruit mesocarp and seeds, and subsequently conducted a series of quantitative real-time polymerase chain reactions (qRT-PCR) at distinct developmental milestones of the fruit. The alignment of qRT-PCR-validated gene expression profiles with those delineated in the RNA-Seq dataset, as illustrated in **Figure 3C**, serves as a compelling affirmation of the sequencing data’s integrity and precision.

### 3.4 Spatiotemporal expression of tissue specific key genes of oil biosynthesis in the mesocarp and seeds

**Figure 4:**
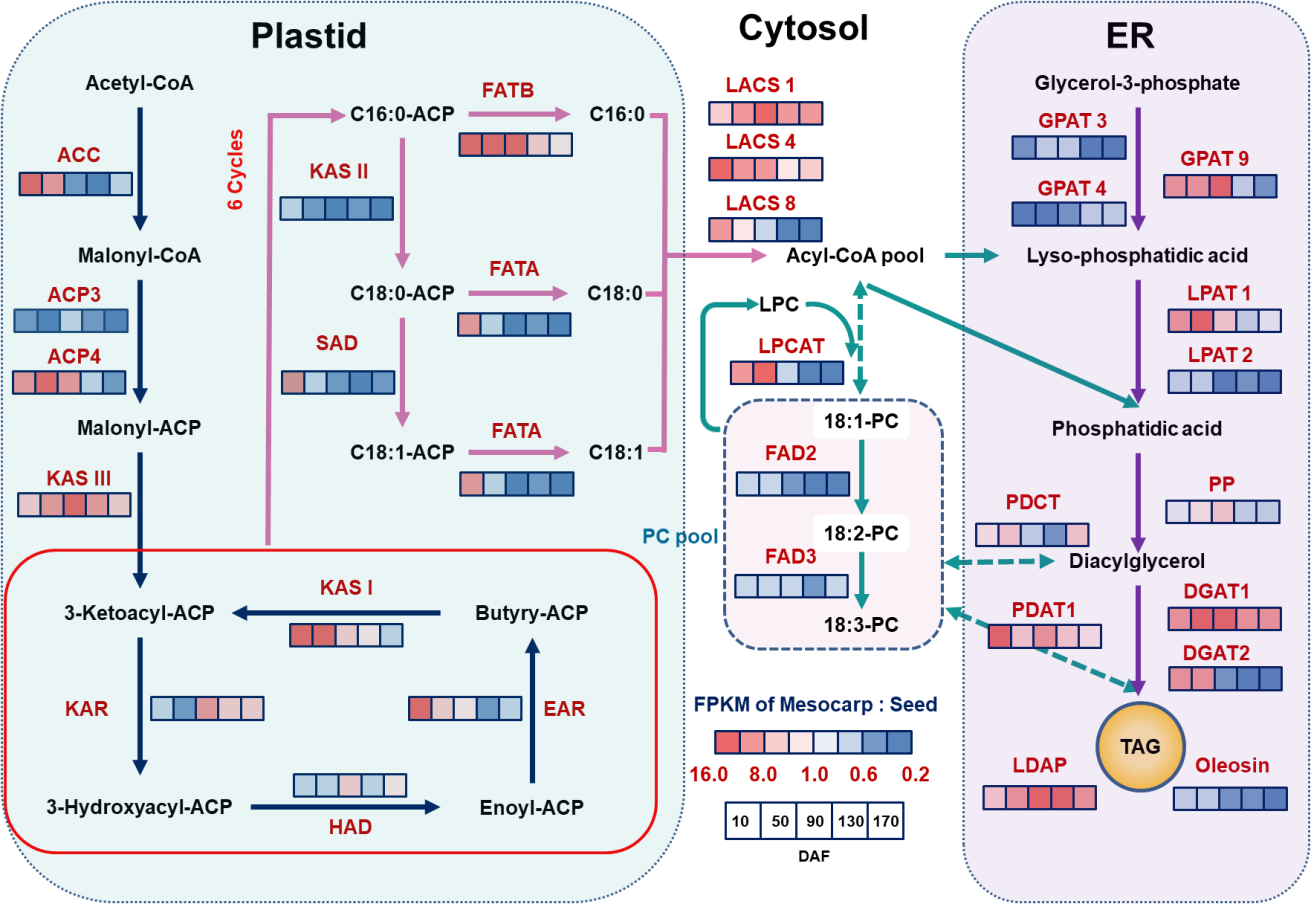
The schematic diagram of the pathway and temporal expressional patterns of oil biosynthesis. The icons close to each enzyme show the results of FPKM normalized ratio value of mesocarp to seeds, from left to right were 10, 50, 90, 130 and 170 DAF. The identified key enzymes involved in lipid metabolism include acetyl-CoA carboxylase carboxyl transferase (ACC); acyl carrier protein (ACP); Malonyl-CoA-ACP transacylase (MAT); 3-Ketoacyl ACP synthase I,II,III (KASI,II,II); 3-Ketoacyl ACP reductase (KAR); 3R-hydroxymyristoyl ACP dehydrase (HAD); enoyl-ACPreductase I (EAR); fatty acyl-ACP thioesterase A (FATA); fatty acyl-ACP thioesterase B (FATB); Stearoyl-ACP desaturase (SAD); long-chain acyl-CoA synthetase (ACSL); fatty acid desaturase (FAD2); glycerol kinase (GK); glycerol-3-phosphate acyltransferase (GPAT); lysophosphatidyl acyltransferase (LPAT); phosphatidate phosphatase (PP); diacylglycerol O-acyltransferase (DGAT); phospholipid: diacylglycerol acyltransferase (PDAT); lysophosphatidylcholine acyltransferase (LPCAT); lipid droplet associated protein (LDAP).Lipid substrates are abbreviated: palmitic acid (16:0); stearic acid (18:0); oleic acid(18:1); linoleic acid(18:2) and linolenic acid (18:3).

Utilizing the functional group annotations provided by the KEGG Pathway, and by integrating the analysis of key enzyme expression levels, specifically the gene expression ratio (FPKM ratio of Mesocarp to Seeds, M/S), our study has identified a distinct specificity in the expression patterns of genes crucial for oil biosynthesis across various regions of the *S. paniculata* fruit, as illustrated in **Figure 4**.

#### 3.4.1 Tissue specific expression of key genes in fatty acid synthesis pathway

In the initial stages of the fatty acid synthesis pathway, acetyl-CoA carboxylase carboxyl transferase (ACC), recognized as the primary rate-limiting enzyme in lipid synthesis, is markedly overexpressed in mesocarp tissue during the early phases of fruit development (10~50 DAF), with an average mesocarp-to-seeds gene expression ratio (M/S) of 14.5. Corroborating this, associated FAS enzyme genes such as Acyl Carrier Protein 4 (ACP4) (M/S of 12), KASIII (M/S of 9.6), KAS I (M/S of 13.5), and Enoyl-ACP reductase (EAR) (M/S of 8.3) mirror these elevated expression levels within the same developmental timeframe. This enriched gene expression in the mesocarp likely initiates lipid synthesis earlier in the fruit’s mesocarp compared to the seeds. This hypothesis is substantiated by the observed lipid accumulation, which is detectable in the mesocarp from 10 DAF, in contrast to seeds, where it commences later at 50 DAF with a comparatively meager quantity (as depicted in **Figure 1**). Furthermore, within the TAG assembly pathway, an analogous trend is observed for genes LACS1 (M/S of 5.7), LACS4 (M/S of 12.2), GPAT9 (M/S of 7.3), DGAT2 (M/S of 9.6), and Phospholipid: Diacylglycerol Acyltransferase 1 (PDAT1) (M/S of 4.0), all of which exhibit increased expression in the mesocarp during the early stages of fruit development (10~50 DAF). These findings reinforce the notion of independent regulatory mechanisms governing the timing and rate of lipid accumulation between the mesocarp and seeds of the fruit.

#### 3.4.2 Tissue specific expression of key genes in fatty acid desaturation pathway

Within the carbon chain elongation and desaturation pathways of fatty acid synthesis, the KAS gene is a pivotal regulator of the carbon chain structure in plant fatty acids. KAS II predominantly catalyzes the synthesis of C18:0-ACP, whereas KAS I is chiefly responsible for C16:0-ACP production. The fatty acyl-ACP thioesterase B (FATB) gene facilitates the release of the ACP moiety from palmitoyl-ACP, yielding palmitic acid. Conversely, the FATA gene catalyzes the release of ACP from 18:0/1-ACP, resulting in stearic acid and C18:0/1. The desaturase SAD promotes the dehydration of C18:0-ACP to C18:1-ACP, and FAD2 further desaturates C18:1-ACP to generate C18:2-ACP. This study revealed that, in seed tissues, the expression of these genes surpasses that in mesocarp tissues, except for the FATB gene. Notably, KAS II (M/S=0.18) and FATA (M/S=0.18) exhibit pronounced expression throughout the fruit’s development (10~170 DAF), potentially contributing to the high prevalence of C18 fatty acids in seed oil, which can reach up to 90%. The desaturases SAD and FAD2 also show elevated expression levels in seed tissues, aligning with the findings in **Figure 1**, where C18:1 and C18:2 fatty acids constitute approximately 50% and 30% of total fatty acids, respectively. The elevated expression of FATA and SAD in the mesocarp at 10 DAF may correlate with the early lipid accumulation observed. In contrast, the FATB gene’s high expression in the mesocarp (M/S=13.6) may enhance the synthesis of the saturated fatty acid C16:0. As depicted in **Figure 1**, palmitic acid content in the mesocarp remains high at maturity (35%), whereas in seeds, it decreases with development, dropping to 10% at maturity. Collectively, the unique expression profiles of the FAT gene family and desaturase enzymes are hypothesized to be the principal determinants of the distinct fatty acid compositions observed in mesocarp and seed tissues.

#### 3.4.3 Tissue specific expression of key genes in TAG assembly pathway

Analyzing the gene expression associated with TAG assembly, we identified tissue-specific expression patterns of isoform genes and subtypes across various regions of the fruit. Notably, LACS1 and LACS4, which are pivotal in converting free fatty acids into acyl-CoA thioesters substrates for TAG synthesis display significant tissue dependency in their expression within the mesocarp, whereas LACS8 is less expressed in seeds. GPAT enzymes, which initiate TAG biosynthesis by transferring acyl groups to glycerol-3-phosphate (G3P) to form lysophosphatidic acid (LPA), exhibit differential expression, with GPAT3 and GPAT4 being highly expressed in seeds (M/S ratios of 0.18 and 0.23, respectively). In contrast, GPAT9 is more abundant in the mesocarp during early development (M/S ratio of 8.5 at 10~90 DAF), with its expression subsequently declining. LPA and acetyl-CoA are transformed into phosphatidic acid (PA) by LPAT, with LPAT1 being preferentially expressed in the mesocarp (M/S ratio of 7.8 at 10~90 DAF) and LPAT2 in seeds (M/S ratio of 0.76 at 10~50 DAF). Diacylglycerol (DAG) is subsequently converted to TAG by diacylglycerol acyltransferase (DGAT), with PDAT1 also contributing to TAG synthesis through the phosphatidylcholine (PC) pathway, as evidenced by its high expression in the mesocarp (M/S value of 5.5). Furthermore, key TAG synthesis enzymes, DGAT1 and DGAT2, are more highly expressed in the mesocarp than in seeds, potentially accounting for the higher lipid accumulation in the mesocarp (approximately 43% of the whole fruit) compared to seeds (about 30%).

#### 3.4.4 Tissue specific expression of key genes in LD related protein

TAG is stored within plant cells as LDs (Horn et al., 2013). The proteins on the LD surface are negatively charged, resulting in steric hindrance and electrostatic repulsion. This contributes to the compartmentalization of LDs, preventing their fusion and ensuring stable storage. Research has shown that expression of lipid droplet associated protein (LDAP) genes is markedly tissue-specific. Oleosin, a key regulator of LD compartmentalization, is highly expressed in seeds, whereas LDAP predominantly controls LDs in the mesocarp. In this study, Oleosin expression was observed to be consistently high throughout fruit development in seeds. Conversely, LDAP was found to be significantly more highly expressed in the mesocarp (M/S ratio of 6.3), with transcription levels in seeds being very low (see **Figure 4**). Observations from **Figure 2** reveal that LDs in white sandalwood seed cells are relatively uniform in morphology and size. In contrast, LDs in mesocarp cells vary in size, with smaller LDs fusing to form larger ones during development. By maturity, a stable state of LDs of different sizes is maintained. These findings further substantiate the distinct regulatory proteins controlling LDs between seed and non-seed tissues. The tissue-specificity of gene expression is thus identified as a primary cause for the variation in lipid synthesis across different tissues.

## 4 Discussion

### 4.1 Tissue specific oil content and composition

Woody oil-bearing plants with whole fruits contain significant amounts of plant oils not only in their seeds but also in other fruit parts, primarily the mesocarp. These plants are diverse and include avocado (*Persea americana*) from the *Lauraceae* family (Ibarra-Laclette et al., 2015), olive (*Olea europaea*) from the Oleaceae (Banilas et al., 2011), oil palm (*Elaeis guineensis*) from the *Arecaceae* (Hassan et al., 2019), *Pistacia chinensis* from the *Anacardiaceae* (Trabelsi et al., 2012), and *Cornus wilsoniana* from the *Cornaceae* (Li et al., 2018). *S. paniculata*, the focus of this study, is among them. Their fruits can be fully utilized, and since the entire fruit contains oil, processing does not require dehulling, simplifying the procedure and reducing production costs.

Furthermore, oil composition variations in different fruit parts can dictate diverse applications. For instance, fresh *Litsea cubeba* mesocarp can yield high-grade aromatic essential oils rich in citral (60% to 90%), while seed oil, rich in lauric acid (50%), is used for high-end health care edible oils (Chen et al., 2020). Oil palm (*Elaeis guineensis*) mesocarp oil, with a high saturation of fatty acids (42% of C16:0), is commonly used in margarine production and extensively in cooking and food manufacturing. In contrast, seed oil, containing a large amount of easily absorbed medium and short-chain fatty acids (70% of MCFA), is ideal for high-value health care edible oils (Barcelos et al., 2015).

Given the considerable differences in TAG accumulation and FA composition across plants and tissues, and considering the constant increase in global demand for vegetable oils used both for food and industrial purposes, the identification of factors regulating these quantitative and qualitative variations is a critical issue, which is the focus of a growing number of studies (Baud and Lepiniec, 2010; Chapman and Ohlrogge, 2012). Our study reveals significant lipid component variations in different parts of *S. paniculata* fruits. The mesocarp contains 42% oil with a balanced ratio of saturated fatty acids (C16:0) to monounsaturated fatty acids (C18:1) to polyunsaturated fatty acids (C18:2) of approximately 1:1:1. The seeds contain 30% oil, 90% of which are unsaturated fatty acids. These differences suggest that oils from various parts of *S. paniculata* fruits possess excellent qualities and potential for diverse applications.

### 4.2 Tissue specific expression of genes in oil biosynthesis

Plant lipid synthesis involves the coordinated expression of numerous enzymes and regulatory genes. Research indicates that most oil-bearing woody plants with whole fruits exhibit similar lipid synthesis pathways in both their seeds and non-seed tissues (Pyc et al., 2017). The primary pathways include fatty acid synthesis and the Kennedy pathway for TAG. The synthesis process generally involves the conversion of acetyl-CoA into fatty acids by acetyl-CoA carboxylase and a series of fatty acid synthase enzymes (FAS). Subsequently, the activated fatty acids as acyl-CoA are transferred to glycerol by acyltransferases GPAT, LPAT, and DGAT in sequence to produce TAG, or PDAT catalyzes the formation of DAG, which is then further synthesized into TAG. Variations in gene transcription levels within the lipid metabolic pathways of different oil-bearing plant species regulate enzyme activities, resulting in disparities in TAG and other lipid component concentrations in various fruit parts, such as the mesocarp and seeds (Bates, 2016).

Our study discovered a temporal discrepancy in lipid synthesis between the fruit mesocarp and seeds of *S. paniculata*. oil accumulation in the mesocarp is observable from 10 DAF, whereas in seeds, it commences from 50 DAF. This difference is reflected in the preferential expression of genes like ACC and ACP4 in the fruit mesocarp tissue during the early stages of fruit development (10 to 50 DAF). Tissue types are subject to distinct regulatory mechanisms (Battey and Ohlrogge, 1990), and during the early fruit development phase, the fruit peel, rich in chloroplasts, particularly induces ACP4, which is sensitive to light responses (Bonaventure and Ohlrogge, 2002). This induction is crucial for meeting the demand for fatty acid biosynthesis directed towards TAG storage in heterotrophic non-seed tissues.

Additionally, the differences in lipid composition are primarily influenced by the differential regulation of genes related to carbon chain elongation and desaturation in fatty acid synthesis. This study found that the high expression of the FATB gene in the fruit mesocarp of *S. paniculata* promotes the synthesis of more C16:0, reaching up to 35% at maturity. In contrast, the KAS II and FATA gene family members are significantly expressed throughout the developmental stages (10~170 DAF) in seeds, which may lead to the total content of C18 fatty acids (such as C18:0, C18:1, C18:2, and C18:3) in seed oil reaching up to 90%. The desaturase enzymes SAD and FAD2 also show higher expression activity in seed tissues. This is consistent with the observation shown in **Figure 1**, where unsaturated fatty acids C18:1 and C18:2 account for approximately 50% and 30% of the total fatty acids (FA), respectively. Similar results are also found in other oil-containing fruit plants. For example, the significant differences in the activity of the KASII and FATB gene family members in the seed and fruit mesocarp tissues of oil palm (*Elaeis guineensis*) led to different fatty acid compositions in palm oil and palm kernel oil, with palm oil being rich in saturated fatty acids and palm kernel oil containing a large amount of lower fatty acids (Dussert et al., 2013). Similarly, the high expression of PaFATB in avocado (*Persea americana*) fruit mesocarp promotes the accumulation of more C16:0, while the upregulation of PaFATA and PaFAD2 genes in seeds is the reason for the higher content of C18 fatty acids in the seeds (Kilaru et al., 2015).

DAG is ultimately transformed into TAG through the catalytic action of DGAT. Additionally, TAG can also be synthesized from PC under the catalysis of PDAT. Our findings reveal that the elevated expression of PDAT1 in *S. paniculata* fruit mesocarp suggests a contribution to TAG synthesis from the PDAT pathway, derived from Phosphatidylcholine (PC), particularly during the early stages of fruit development. Moreover, the key TAG synthesis enzymes, DGAT1 and DGAT2, exhibit significantly higher expression levels in the fruit mesocarp compared to seeds, potentially explaining the higher lipid accumulation in the fruit mesocarp (approximately 43% of the whole fruit) versus seeds (about 30%). Similar findings in avocado (*Persea americana*) fruit mesocarp have shown that both PaPDAT1 and PaDGAT1 are highly expressed, suggesting that the PDAT pathway significantly supplements lipid accumulation in avocado (*Persea americana*) fruit mesocarp (Troncoso-Ponce et al., 2011). In the fruit mesocarp of oil palm (*Elaeis guineensis*), genes such as EgDGAT1 and EgDGAT2 are notably more expressed than in seeds, accounting for the fruit mesocarp’s accumulation of approximately 80~90% of the total TAG content of the fruit. In contrast to avocado (*Persea americana*), the reduced expression of EgPDAT1 in oil palm (*Elaeis guineensis*) fruit mesocarp confirms that TAG synthesis primarily relies on the acyl-CoA-dependent DGAT pathway, rather than the PDAT pathway dependent on PC (Dussert et al., 2013).

### 4.3 Tissue specific regulation of lipid droplet proteins

In woody oil-bearing plants, the terminal product of lipid biosynthesis, TAG, buds within the endoplasmic reticulum as LD to form compartmentalized structures, facilitating their long-term storage in the cytoplasm’s aqueous environment. Although lipid droplets in different fruit parts are wrapped by a phospholipid monolayer around a core of neutral lipids, their compartmentalized morphology and function exhibit tissue specificity (Henne et al., 2018). This study observed uniform lipid droplet morphology and size in *S. paniculata* seed cells, whereas fruit mesocarp cells contained lipid droplets of varying sizes that fuse to form larger ones during development. However, at fruit maturity, lipid droplets of different sizes in the mesocarp cells remain stable (**Figure 2**). Lipid droplets in seeds primarily provide energy for germination and seedling growth, while those in the mesocarp serve for insulation, cold protection, and long-term storage (Huang and Huang, 2017).

The variation in lipid droplet compartmentalization is fundamentally attributed to differences in the regulatory mechanisms of lipid droplet formation proteins across various fruit tissues. Our study observed that Oleosin, a crucial lipid droplet-associated protein in seeds, maintains a higher expression level throughout fruit development. It features a hydrophobic central domain that facilitates its integration into the endoplasmic reticulum, where it aids in the formation and stabilization of nascent lipid droplets (Xu et al., 2023). In contrast, LDAP exhibits significantly higher expression in the mesocarp (**Figure 4**). These lipid droplets are strictly regulated by proteins on the endoplasmic reticulum, budding to form uniformly sized droplets once they reach a predetermined size (Henne et al., 2020). The absence of Oleosin has been linked to the fusion of lipid droplets into larger ones, thereby limiting oil accumulation in seed cells (Tsai et al., 2015).

LDAP, a protein identified in non-seed tissues like fruit mesocarp, differs from Oleosin by specifically targeting the surface of lipid droplets as a non-integral protein. It interacts with other proteins to regulate lipid droplet compartmentalization, influencing oil content and fatty acid composition (Pyc et al., 2017). Overexpression of LDAP in tobacco has been shown to increase both the number of lipid droplets and oil content, along with a significant rise in the relative levels of C18:2 and C18:3 (Horn et al., 2013). Furthermore, the absence of LDAP leads to the fusion of lipid droplets in the cytoplasm, forming larger droplets (Coulon et al., 2020).

## 5 Conclusion

This study delineates lipid biosynthesis in S. paniculata, contrasting mesocarp and seed lipid profiles. Mesocarp lipid biosynthesis commences 40 days prior to seeds, peaking at 43% lipid content versus seeds’ 30%. The mesocarp is enriched with palmitic acid (33% of total fatty acids), while seeds are dominated by oleic and linoleic acids (90% unsaturated). Microscopy reveals lipids localized in mesocarp OC and LD, and intracellular LD in seeds, with mesocarp LD size heterogeneity suggesting droplet fusion, unlike the uniform size in seeds. Gene expression profiling distinguished mesocarp and seed tissues in *S. paniculata*. Light-responsive genes ACP4, ACC, and EAR were predominantly upregulated in the mesocarp, initiating lipid synthesis earlier than in seeds. The mesocarp’s elevated expression of FATA facilitated palmitic acid synthesis, while PDAT was crucial for the early stages of TAG synthesis. Tissue-specific upregulation of TAG assembly genes LACS1/4, GPAT9, LPAT1, and DGAT1/2 correlated with the mesocarp’s higher lipid content. In seeds, KAS II, FATA, SAD, and FAD2 were highly expressed, contributing to increased C18 fatty acid content. GPAT3/4, LACS8, and LPAT2 also showed seed-specific expression. Oleosin was more highly expressed in seeds, whereas LDAP was more abundant in the mesocarp. These findings underscore the role of tissue-specific gene regulation in lipid synthesis variability and identify key genes and pathways for improving lipid quality and yield in *S. paniculata*.

## Data Availability

The datasets presented in this study can be found in online repositories. The names of the repository/repositories and accession number(s) can be found below: https://www.ncbi.nlm.nih.gov/, SRA357712.
