# Supplementary material for: Comparative analysis of transcriptome in oil biosynthesis between seeds and non-seed tissues of Symplocos paniculata fruit
Source: Front Plant Sci. 2024 Oct 2;15:1441602. doi: 10.3389/fpls.2024.1441602 (PMC11479902; doi:10.3389/fpls.2024.1441602)
Supplement: Supplementary file 1 [file DataSheet1.docx]

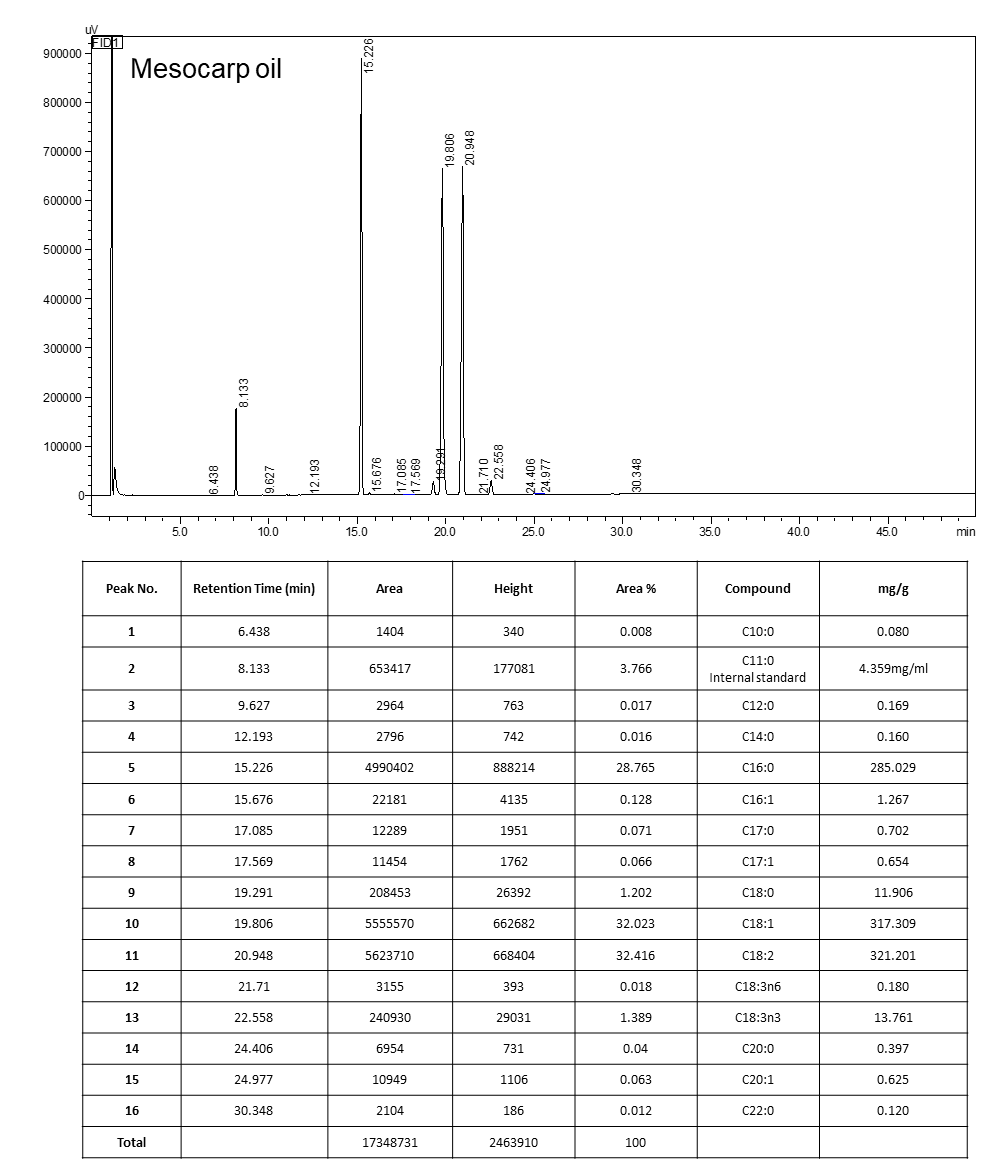


Figure S1 The mass spectrometry for mesocarp oil fatty acid components of *Symplocos paniculate*


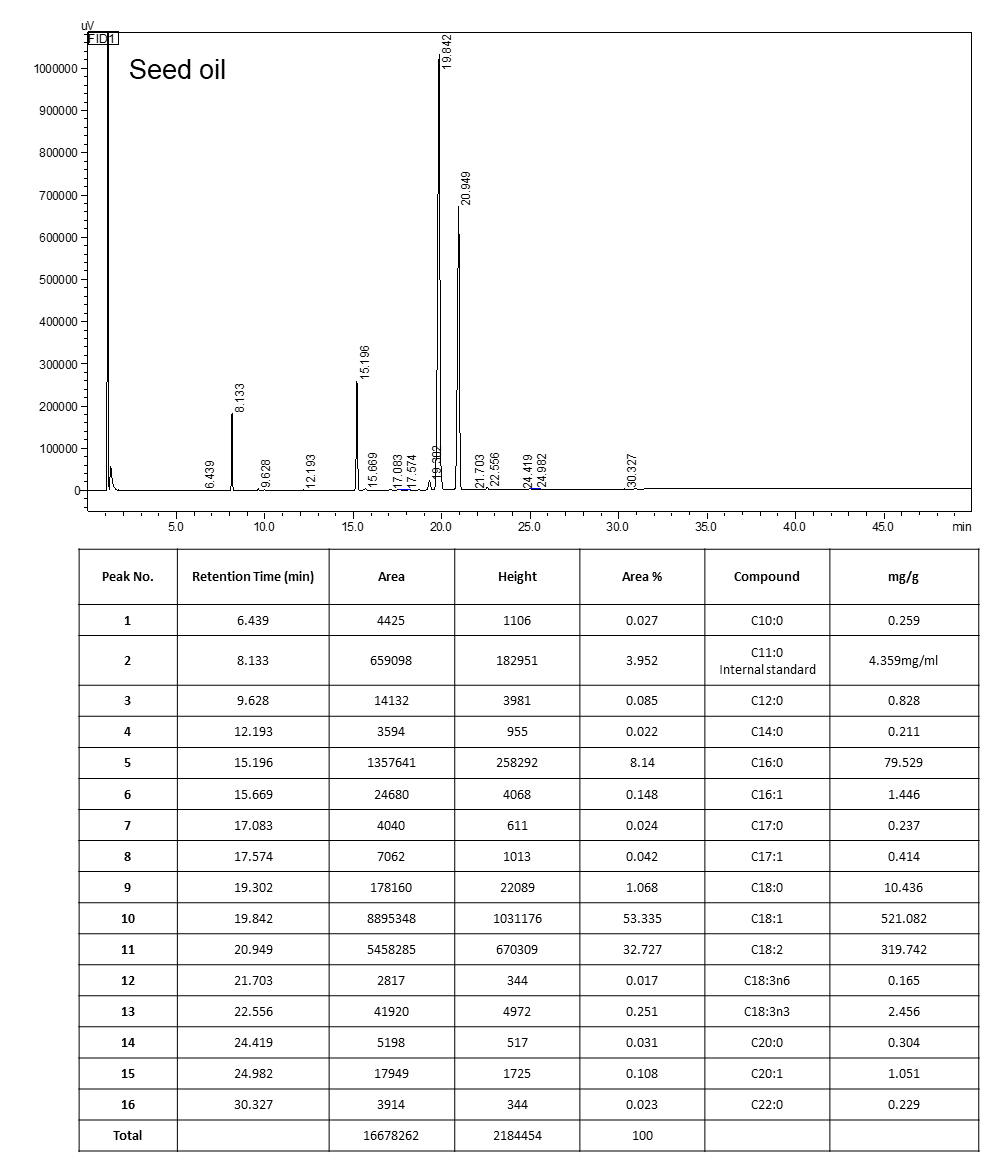


Figure S2 The mass spectrometry for mesocarp oil fatty acid components of *Symplocos paniculate*


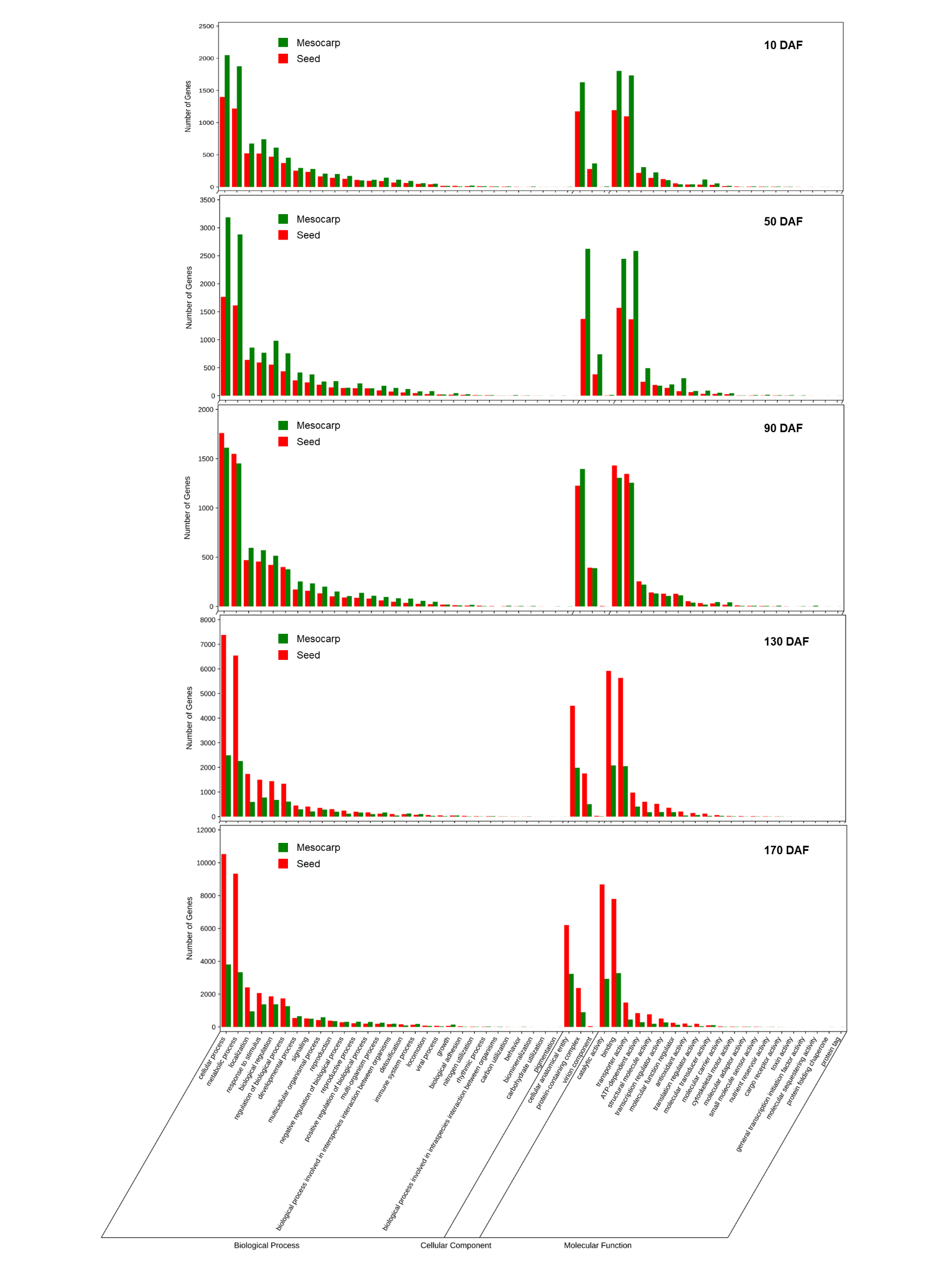
Figure S3 Comparative GO classification of unigenes in mesocarp and seed tissues


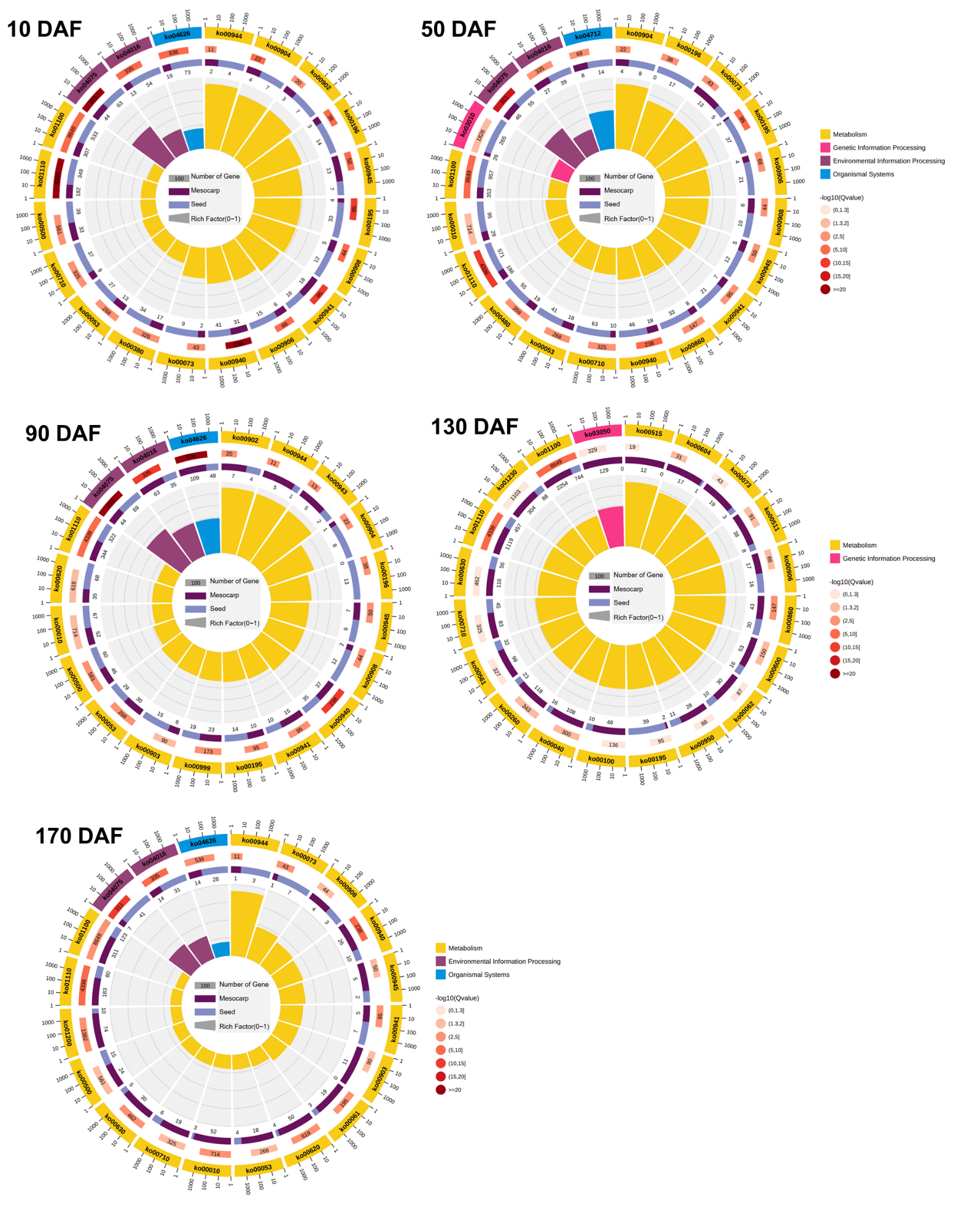


Figure S4 KEGG pathway analysis of unigene functional annotation in mesocarp and seed tissues

Table S1. The designed primers of the key enzymes involved in lipid metabolism for qRT-PCR

| **Unigene ID** | **Fruit tissue** | **Putative function** | **Abbreviation** | **Forward (5'- 3')** | **Reverse (5'-3')** | **PCR products (bp)** |
| --- | --- | --- | --- | --- | --- | --- |
| Unigene112167 | Mesocarp | Acetyl-CoA Carboxylase | ACC | TACAACGCAGGCATCAGA | GGCAAGTTTCACCGCACA | 98 |
| Unigene82378 | Seed | Acetyl-CoA Carboxylase | ACC | CGGTTAGGGCACTTGACG | TCGCTCCAGGCAGGGTAT | 237 |
| Unigene112447 | Mesocarp | Phospholipid:Diacylglycerol Acyltransferase1 | PDAT1 | TGCGGGAATACAAACATA | CTCCGACATCCTCATCAC | 175 |
| Unigene112820 | Seed | Phospholipid:Diacylglycerol Acyltransferase1 | PDAT1 | CTTGAGGGTTTGGATGCT | AATGGAGGGATGTTGCTG | 114 |
| Unigene54346 | Mesocarp | Acyl-CoA:Diacylglycerol Acyltransferase2 | DGAT2 | GTTGGAGCATCCTCGTCT | GGTGCCTTTACTGATTGTG | 361 |
| Unigene114377 | Seed | Acyl-CoA:Diacylglycerol Acyltransferase2 | DGAT2 | CAACAAGGACGGAGACGC | CGGGCATTGCTCAAGATC | 143 |
